# Supplementary material for: IGSF10 mutations dysregulate gonadotropin‐releasing hormone neuronal migration resulting in delayed puberty
Source: EMBO Mol Med. 2016 Apr 13;8(6):626–42. doi: 10.15252/emmm.201606250 (PMC4888853; doi:10.15252/emmm.201606250)
Supplement: Supplementary file 1 — Appendix [file EMMM-8-626-s001.pdf]

Appendix - *IGSF10* regulates embryonic GnRH neuronal migration and mutations result in delayed puberty

Howard SR, Guasti L, Ruiz-Babot G, Mancini A, David A, Storr HL, Metherell LA, Sternberg MJE, Cabrera CP, Warren HR, Barnes MR, Quinton R, de Roux N, Young J, Guiochon-Mantel A, Wehkalampi K, André V, Gothilf Y, Cariboni A, Dunkel L

Table of Contents

Appendix Figure S1: Multiple sequence alignment (m.s.a.) for all four residues harboring *IGSF10* mutations.

Appendix Figure S2: Tertiary structure of LRR Region I.

Appendix Figure S3: Efficacy of Sp-MO.

Appendix Table S1: Rare Variant Burden Testing post Targeted Exome Sequencing

Appendix Table S2: Auxological and Pubertal Data of DP patients from the cohort with potentially pathogenic mutations in *IGSF10* as compared to patients without mutations in *IGSF10.*

Appendix Table S3: Frequency of *IGSF10* variants in the hypogonadotropic hypogonadism (HH) cohort.

Appendix Table S4: Clinical characteristics of two patients with hypothalamic amenorrhea (HA) and HA-equivalent with a shared rare variant in *IGSF10* (NM_178822: c.7353_7354insATCA: (rs570110855) p.L2452fs).

A

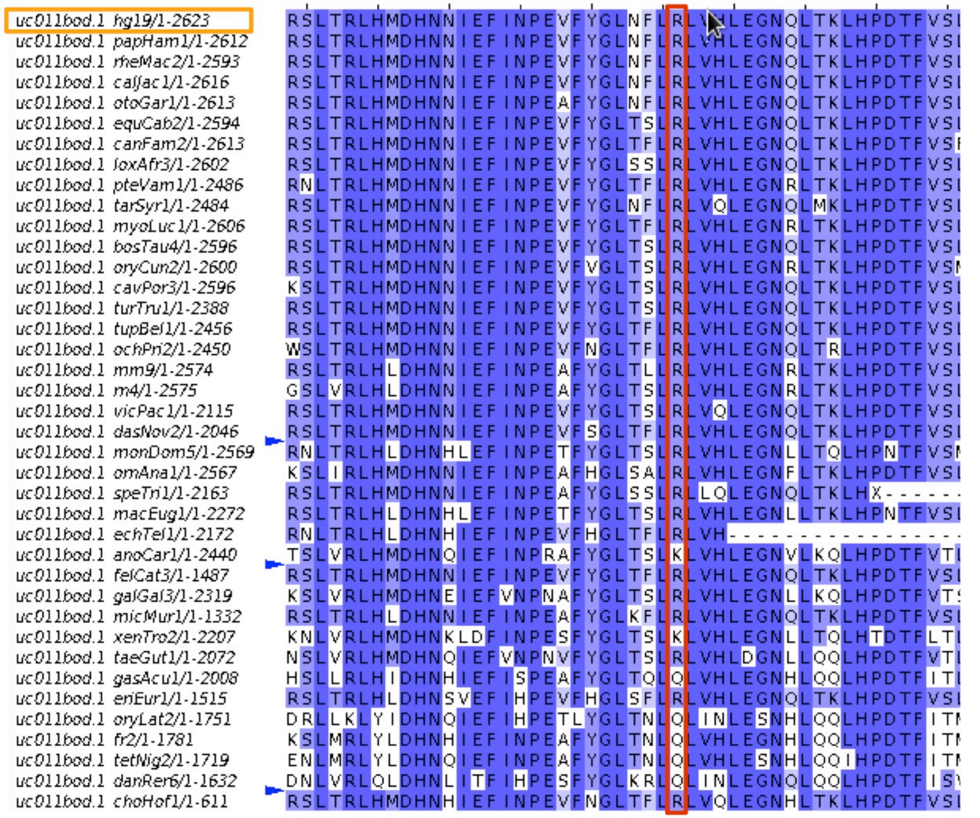
uc011bod.1 hg19/1-2623
uc011bod.1 papHam1/1-2612
uc011bod.1 rheMac2/1-2593
uc011bod.1 calJac1/1-2616
uc011bod.1 otoGar1/1-2613
uc011bod.1 equCab2/1-2594
uc011bod.1 canFam2/1-2613
uc011bod.1 loxAfr3/1-2602
uc011bod.1 pteVam1/1-2486
uc011bod.1 tarSyr1/1-2484
uc011bod.1 myoLuc1/1-2606
uc011bod.1 bosTau4/1-2596
uc011bod.1 oryCun2/1-2600
uc011bod.1 cavPor3/1-2596
uc011bod.1 turTru1/1-2388
uc011bod.1 tupBel1/1-2456
uc011bod.1 ochPri2/1-2450
uc011bod.1 mm9/1-2574
uc011bod.1 rn4/1-2575
uc011bod.1 vicPac1/1-2115
uc011bod.1 dasNov2/1-2046
uc011bod.1 monDom5/1-2569
uc011bod.1 ornAna1/1-2567
uc011bod.1 speTri1/1-2163
uc011bod.1 macEug1/1-2272
uc011bod.1 echTel1/1-2172
uc011bod.1 anoCar1/1-2440
uc011bod.1 felCat3/1-1487
uc011bod.1 galGal3/1-2319
uc011bod.1 micMur1/1-1332
uc011bod.1 xenTro2/1-2207
uc011bod.1 taeGut1/1-2072
uc011bod.1 gasAcu1/1-2008
uc011bod.1 eriEur1/1-1515
uc011bod.1 oryLat2/1-1751
uc011bod.1 fr2/1-1781
uc011bod.1 tetNig2/1-1719
uc011bod.1 danRer6/1-1632
uc011bod.1 choHof1/1-611

B

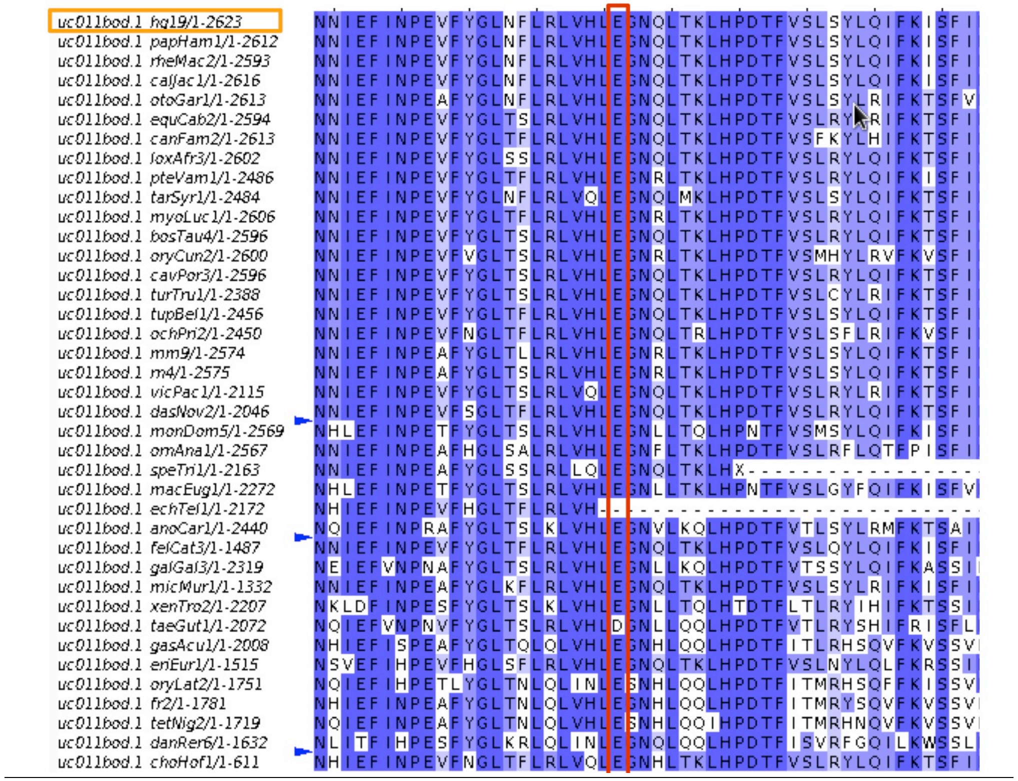

uc011bod.1 hg19/1-2623
uc011bod.1 papHam1/1-2612
uc011bod.1 rheMac2/1-2593
uc011bod.1 calJac1/1-2616
uc011bod.1 otoGar1/1-2613
uc011bod.1 equCab2/1-2594
uc011bod.1 canFam2/1-2613
uc011bod.1 loxAfr3/1-2602
uc011bod.1 pteVam1/1-2486
uc011bod.1 tarSyr1/1-2484
uc011bod.1 myoLuc1/1-2606
uc011bod.1 bosTau4/1-2596
uc011bod.1 oryCun2/1-2600
uc011bod.1 cavPor3/1-2596
uc011bod.1 turTru1/1-2388
uc011bod.1 tupBel1/1-2456
uc011bod.1 ochPri2/1-2450
uc011bod.1 mm9/1-2574
uc011bod.1 rn4/1-2575
uc011bod.1 vicPac1/1-2115
uc011bod.1 dasNov2/1-2046
uc011bod.1 monDom5/1-2569
uc011bod.1 ornAna1/1-2567
uc011bod.1 speTri1/1-2163
uc011bod.1 macEug1/1-2272
uc011bod.1 echTel1/1-2172
uc011bod.1 anoCar1/1-2440
uc011bod.1 felCat3/1-1487
uc011bod.1 galGal3/1-2319
uc011bod.1 micMur1/1-1332
uc011bod.1 xenTro2/1-2207
uc011bod.1 taeGut1/1-2072
uc011bod.1 gasAcu1/1-2008
uc011bod.1 eriEur1/1-1515
uc011bod.1 oryLat2/1-1751
uc011bod.1 fr2/1-1781
uc011bod.1 tetNig2/1-1719
uc011bod.1 danRer6/1-1632
uc011bod.1 choHof1/1-611

C

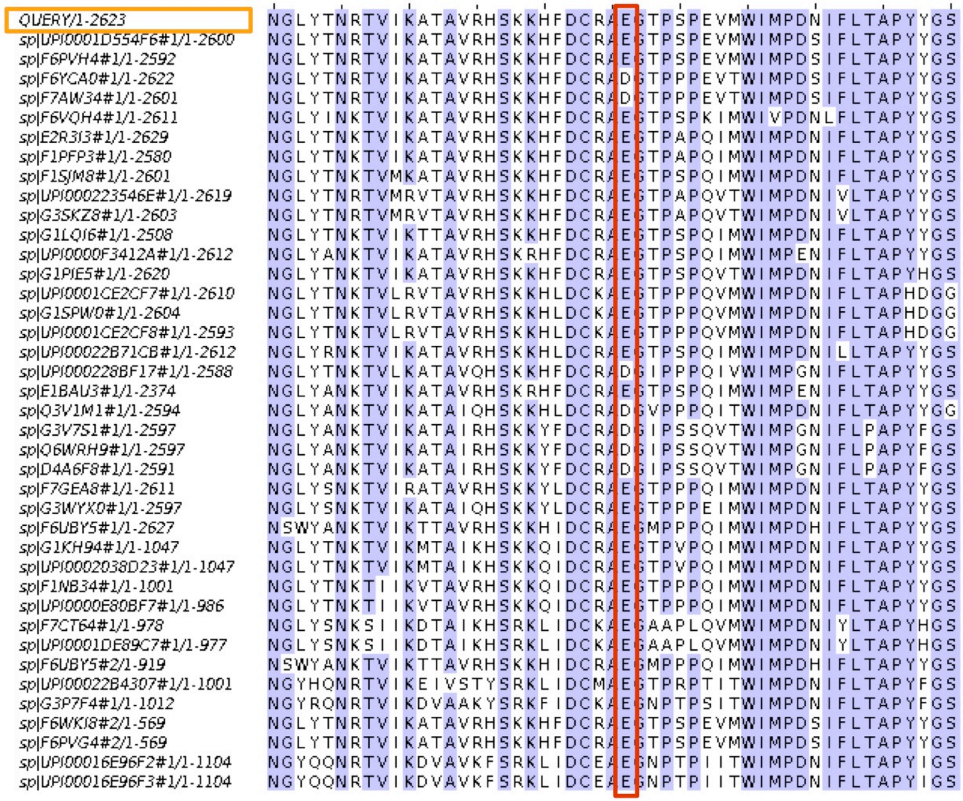
QUERY/1-2623 NGLYTNRTVIKATAVRHSKKHFDCRAEGTPSPEVMWIMPDNIFLTAPYYGS
sp|UPI0001D554F6#1/1-2600 NGLYTNRTVIKATAVRHSKKHFDCRAEGTPSPEVMWIMPDSIFLTAPYYGS
sp|F6PVH4#1/1-2592 NGLYTNRTVIKATAVRHSKKHFDCRAEGTPSPEVMWIMPDSIFLTAPYYGS
sp|F6YCA0#1/1-2622 NGLYTNRTVIKATAVRHSKKHFDCRADGTPPPEVTWIMPDSIFLTAPYYGS
sp|F7AW34#1/1-2601 NGLYTNRTVIKATAVRHSKKHFDCRADGTPPPEVTWIMPDSIFLTAPYYGS
sp|F6VQH4#1/1-2611 NGLYINKTVIKATAVRHSKKHFDCRAEGTPSPKIMWIVPDNLFLTAPYYGS
sp|E2R3I3#1/1-2629 NGLYTNKTVIKATAVRHSKKHFDCRAEGTPAPQIMWIMPDNIFLTAPYYGS
sp|F1PFP3#1/1-2580 NGLYTNKTVIKATAVRHSKKHFDCRAEGTPAPQIMWIMPDNIFLTAPYYGS
sp|F1SJM8#1/1-2601 NGLYTNKTVMKATAVRHSKKHFDCRAEGTPSPQIMWIMPDNIFLTAPYYGS
sp|UPI000223546E#1/1-2619 NGLYTNRTVMRVTAVRHSKKHFDCRAEGTPAPQVTWIMPDNIVLTAPYYGS
sp|G3SKZ8#1/1-2603 NGLYTNRTVMRVTAVRHSKKHFDCRAEGTPAPQVTWIMPDNIVLTAPYYGS
sp|G1LQI6#1/1-2508 NGLYTNKTVIKTTAVRHSKKHFDCRAEGTPSPQIMWIMPDNIFLTAPYYGS
sp|UPI0000F3412A#1/1-2612 NGLYANKTVIKATAVRHSKRHFDCRAEGTPSPQIMWIMPENIFLTAPYYGS
sp|G1PIE5#1/1-2620 NGLYTNKTVIKATAVRHSKKHFDCRAEGTPSPQVTWIMPDNIFLTAPYHGS
sp|UPI0001CE2CF7#1/1-2610 NGLYTNKTVLRVTAVRHSKKHLDCKAEGTPPPQVMWIMPDNIFLTAPHDGG
sp|G1SPW0#1/1-2604 NGLYTNKTVLRVTAVRHSKKHLDCKAEGTPPPQVMWIMPDNIFLTAPHDGG
sp|UPI0001CE2CF8#1/1-2593 NGLYTNKTVLRVTAVRHSKKHLDCKAEGTPPPQVMWIMPDNIFLTAPHDGG
sp|UPI00022B71CB#1/1-2612 NGLYRNKTVIKATAVRHSKKHLDCRAEGTPSPQIMWIMPDNILLTAPYYGS
sp|UPI000228BF17#1/1-2588 NGLYTNKTVLKATAVQHSKKHFDCRADGIPPPQIVWIMPGNIFLTAPYYGS
sp|E1BAU3#1/1-2374 NGLYANKTVIKATAVRHSKRHFDCRAEGTPSPQIMWIMPENIFLTAPYYGS
sp|Q3V1M1#1/1-2594 NGLYANKTVIKATAIQHSKKHLDCRADGVPPPQITWIMPDNIFLTAPYYGG
sp|G3V7S1#1/1-2597 NGLYANKTVIKATAIRHSKKYFDCRADGIPSSQVTWIMPGNIFLPAPYFGS
sp|Q6WRH9#1/1-2597 NGLYANKTVIKATAIRHSKKYFDCRADGIPSSQVTWIMPGNIFLPAPYFGS
sp|D4A6F8#1/1-2591 NGLYANKTVIKATAIRHSKKYFDCRADGIPSSQVTWIMPGNIFLPAPYFGS
sp|F7GEA8#1/1-2611 NGLYSNKTVIRATAVRHSKKYLDCRAEGTPPPQIMWIMPDNIFLTAPYYGS
sp|G3WYX0#1/1-2597 NGLYSNKTVIKATAIQHSKKYLDCRAEGTPPPEIMWIMPDNIFLTAPYYGS
sp|F6UBY5#1/1-2627 NSWYANKTVIKTTAVRHSKKHIDCRAEGMPPPQIMWIMPDHIFLTAPYYGS
sp|G1KH94#1/1-1047 NGLYTNKTVIKMTAIKHSKKQIDCRAEGTPVPQIMWIMPDNIFLTAPYYGS
sp|UPI0002038D23#1/1-1047 NGLYTNKTVIKMTAIKHSKKQIDCRAEGTPVPQIMWIMPDNIFLTAPYYGS
sp|F1NB34#1/1-1001 NGLYTNKTIIKVTAVRHSKKQIDCRAEGTPPPQIMWIMPDNIFLTAPYYGS
sp|UPI0000E80BF7#1/1-986 NGLYTNKTIIKVTAVRHSKKQIDCRAEGTPPPQIMWIMPDNIFLTAPYYGS
sp|F7CT64#1/1-978 NGLYSNKSIIKDTAIKHSRKLIDCKAEGAAPLQVMWIMPDNIYLTAPYHGS
sp|UPI0001DE89C7#1/1-977 NGLYSNKSIIKDTAIKHSRKLIDCKAEGAAPLQVMWIMPDNIYLTAPYHGS
sp|F6UBY5#2/1-919 NSWYANKTVIKTTAVRHSKKHIDCRAEGMPPPQIMWIMPDHIFLTAPYYGS
sp|UPI00022B4307#1/1-1001 NGYHQNRTVIKEIVSTYSRKLIDCMAEGTPRPTITWIMPDNIFLTAPYFGS
sp|G3P7F4#1/1-1012 NGYRQNRTVIKDVAAKYSRKFIDCKAEGNPTPSITWIMPDNIFLTAPYFGS
sp|F6WKI8#2/1-569 NGLYTNRTVIKATAVRHSKKHFDCRAEGTPSPEVMWIMPDSIFLTAPYYGS
sp|F6PVG4#2/1-569 NGLYTNRTVIKATAVRHSKKHFDCRAEGTPSPEVMWIMPDSIFLTAPYYGS
sp|UPI00016E96F2#1/1-1104 NGYQQNRTVIKDVAVKFSRKLIDCEAEGNPTPIITWIMPDNIFLTAPYIGS
sp|UPI00016E96F3#1/1-1104 NGYQQNRTVIKDVAVKFSRKLIDCEAEGNPTPIITWIMPDNIFLTAPYIGS

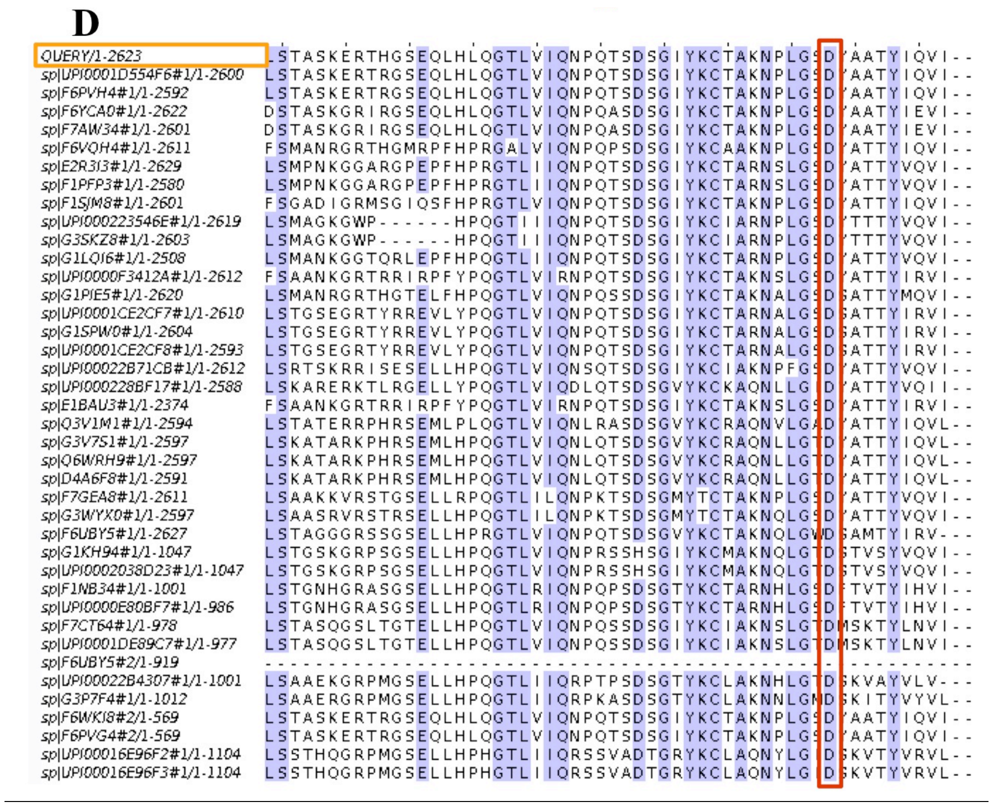

Appendix Figure S1

Multiple sequence alignment (m.s.a.) for all four residues harboring *IGSF10* mutations.

M.s.a. for residues R156 (A), E161 (B), E2264 (C) and D2614 (D). M.sa. was generated using Polyphen2 version 2.2.2, which retrieves homologues (both orthologs and paralogs, here displayed according to their Uniprot Id) using Blast+. M.s.a. is dislayed using Jalview applet. Residues are colored according to the percentage of the residues in each column that agree with the consensus sequence (percentage identity color scheme in Jalview, dark blue >80%, white < 40%).

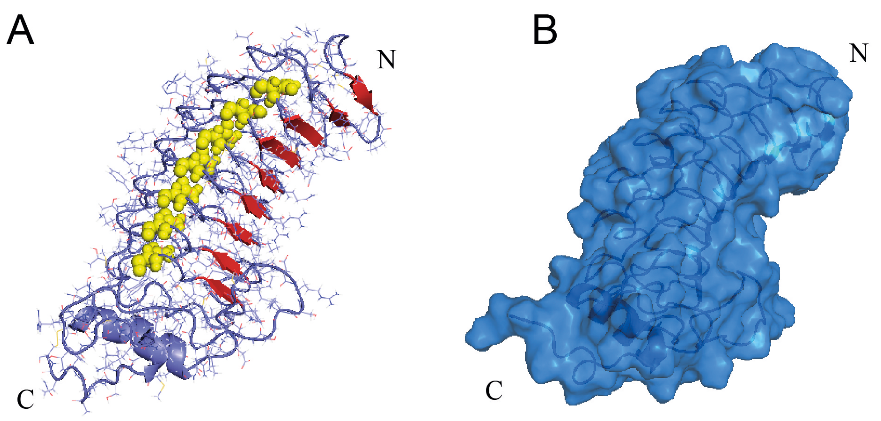

Appendix Figure S2

Tertiary structure of LRR Region I.

Panel A: Beta strands are displayed in red and the asparagine ladder in yellow. Panel B: the ‘arc’ shape of LRR region I.

```
Mispair    GGAGACATCCAGACAGCTGTGGAAAGAATTAACCTGGGGTATA-----------------
Sp-MO      GGAGACATCCAGACAGCTGTGGAAAGAATTAACCTGGGGTAAAACCTACAACAGGCAAAC
           ***************************************** *

Mispair    ------------------------------------------------------------
Sp-MO      CACCGCTGAAATTTCTCGAGAAATGCAATCGAAACTGCTTACTTGAGCTCAGTTCCTCTG

Mispair    -----------------------ACAGTTTGTCAAGTCTTCAAGCGAACGSTCTCTYGGG
Sp-MO      TGCAATTTTGTGTTTCAGGTATAACAGTTTGTCAAGTCTTCAAGCGAACGnTCTCTNGGG
                                  *************************** ***** ***

Mispair    ACTGAATAAGCTGGAAYTGTTAATGCTGCATAGTAACATGATCAAAACTGTnGAGGACAG
Sp-MO      ACTGAATAAGCTGGAANTGTTAATGCTGCATAGTAACATGATCAAAACTGTnGAGGACAG
           **************** *******************************************
```

Appendix Figure S3

Efficacy of Sp-MO.

Nucleotide sequence obtained from RNA of *Igsf10* Sp-MO injected embryos showing an insertion of 97 nucleotides in the splice site of the second exon. Apostrophes denote homology.

Appendix Table S1: Rare Variant Burden Testing post Targeted Exome Sequencing

Gene	Total number of rare (MAF <2.5%) and predicted damaging* variant alleles in probands and in the reference population		P value	Adjusted P value**
	In DP cohort (n=49)	In ExAC Finn database (n=3305)		
**HS6ST1**	4	3	3.23E-06	3.01E-05
**LRRIQ3**	12	88	1.66E-07	4.65E-06
**EAP1**	8	39	1.42E-06	1.99E-05
**IGSF10**	13	292	0.0029	0.020
**SEC24A**	3	17	0.0051	0.029
**ZNF560**	2	6	0.00823	0.038
**FTO**	3	29	0.0188	0.058
**LGR4**	8	172	0.0155	0.058
**CYFIP1**	2	10	0.01852	0.058
**TTF1**	7	96	0.023	0.064
**THBS4**	3	33	0.02574	0.066
**INHA**	3	39	0.0384	0.090
**SYPL2**	5	114	0.04472	0.096
**ELFN2**	2	22	0.06745	0.135
**SETBP1**	2	25	0.0829	0.155
**TBX6**	4	102	0.1204	0.211

**FANCL**	3	78	0.175	0.288
**ZNF266**	2	54	0.236	0.367
**HTR3D**	2	60	0.3035	0.433
**ICAM3**	1	20	0.315	0.433
**BCLAF1**	4	393	0.3249	0.433
**ARHGEF16**	3	100	0.4329	0.551
**CUX1**	1	39	0.5141	0.626
**CPZ**	2	196	0.5867	0.684
**PANX3**	2	120	1	1
**WDR25**	2	126	1	1
**ERCC4**	3	202	1	1
**LRRN1**	0	4	1	1

Minor allele frequency (MAF) data was retrieved from the ExAC Browser (Exome Aggregation Consortium (ExAC), Cambridge, MA: http://exac.broadinstitute.org, accessed October 2015). *by both SIFT (1) and Polyphen2 (2). P value calculated by Fisher's exact test. **Benjamini & Hochberg method (3)

Appendix Table S2: Auxological and Pubertal Data of DP patients from the cohort with potentially pathogenic mutations in *IGSF10* as compared to patients without mutations in *IGSF10.*

	**Males**		
	DP patients with *IGSF10* mutations (mean±SD)	DP patients without *IGSF10* mutations (mean±SD)	
	n=18	n=64	
**Birth weight (kg)**	3.6±0.6	3.7±0.4	ns
**Birth length (cm)**	50.6±1.9	50.9±1.2	ns
**Age at G2 stage (yrs)**	15.2±0.8	15.2±0.9	ns
**Age at "take off" (yrs)**	15.0±1.3	14.9±0.7	ns
**Age at PHV (yrs)**	16.3±1.2	16.1±1.0	ns
**Adult height (cm)**	177.0±5.9	179.3±7.0	ns
	**Females**		
	DP patients with *IGSF10* mutations (mean±SD)	DP patients without *IGSF10* mutations (mean±SD)	
	n=8	n=125	
**Birth weight (kg)**	3.7±0.2	3.6±0.5	ns
**Birth length (cm)**	50.3±2.3	50.0±2.2	ns
**Age at B2 stage (yrs)**	13.5±1.0	13.8±1.0	ns
**Age at "take off" (yrs)**	13.3±0.7	12.5±1.1	ns
**Age at PHV (yrs)**	14.5±0.7	13.4±1.0	ns
**Adult height (cm)**	168.5±5.4	165.1±6.5	ns

Tables are divided into male and female and compare auxological data and pubertal timing of those DP patients with potentially pathogenic mutations in *IGSF10* (p.Arg156Leu, p.Glu161Lys, p.Glu2264Gly and p.Asp2614Asn) with those without mutations in *IGSF10.* All comparisons were found to be non-significant (ns) by Student's *t* test (2-tailed); G2 – Tanner genital stage 2; B2 – Tanner breast stage 2; "take off" – age at start of pubertal growth spurt; PHV – peak height velocity.

Appendix Table S3: Frequency of *IGSF10* variants in the hypogonadotropic hypogonadism (HH) cohort.

**HH cohort (n=334)**			**ExAC database (n=32200)**			**P value**
Total number of rare and predicted damaging variant alleles*	Allele Frequency	Total Prevalence	Total number of rare and predicted damaging variant alleles*	Allele Frequency	Total Prevalence	
34	5.1%	10.2%	2314	3.5%	7%	0.0329

Minor allele frequency (MAF) data for the European control subjects was retrieved from the ExAC Browser (Exome Aggregation Consortium (ExAC), Cambridge, MA: http://exac.broadinstitute.org, accessed October 2015). P value calculated by Fisher's exact test. *rare variants with MAF <2.5% and predicted damaging by both SIFT and Polyphen2

Appendix Table S4: Clinical characteristics of two patients with hypothalamic amenorrhea (HA) and HA-equivalent with a shared rare variant in *IGSF10* (NM_178822: c.7353_7354insATCA: (rs570110855) p.L2452fs).

Characteristic	Patient 1	Patient 2
Clinical Characteristics		
Gender	Female	Male
Age (yr)		
At menarche	Unknown	-
At diagnosis	25	29
BMI at diagnosis	25 (low body fat to muscle ratio)	18
Predisposing factors		
Weight loss	No	Yes
Excessive exercise	Yes	No
Subclinical eating disorder	No	Yes
Fertility Status	Failed attempt at conception off treatment; undergoing gonadotropin therapy	No attempt at conception
Family history of hypothalamic amenorrhea	No	No
Radiological Findings		
MRI	Normal pituitary	Normal pituitary
Biochemical Findings		
LH (U/L)	5.0	0.6
FSH (U/L)	5.9	4.1
Estradiol (E_2) (pg/mL)	<15	-
Testosterone (ng/mL)	<0.3	0.8
Prolactin (mU/L)	129	111
TFTs	Within normal range	Within normal range
AMH	40.3	-

The body-mass index (BMI) is the weight in kilograms divided by the square of the height in meters; LH- lutenising hormone; FSH – follicular stimulating hormone; TFTs – thyroid function tests, comprising of free T4 and thyroid stimulating hormone; AMH – anti-mullerian hormone

References

1. Kumar P, Henikoff S, and Ng PC. Predicting the effects of coding non-synonymous variants on protein function using the SIFT algorithm. *Nature protocols.* 2009;4(7):1073-81.
2. Adzhubei IA, Schmidt S, Peshkin L, Ramensky VE, Gerasimova A, Bork P, Kondrashov AS, and Sunyaev SR. A method and server for predicting damaging missense mutations. *Nature methods.* 2010;7(4):248-9.
3. Benjamini Y, Drai D, Elmer G, Kafkafi N, and Golani I. Controlling the false discovery rate in behavior genetics research. *Behav Brain Res.* 2001;125(1-2):279-84.
