# Supplementary material for: IGSF10 mutations dysregulate gonadotropin‐releasing hormone neuronal migration resulting in delayed puberty
Source: EMBO Mol Med. 2016 Apr 13;8(6):626–42. doi: 10.15252/emmm.201606250 (PMC4888853; doi:10.15252/emmm.201606250)
Supplement: Supplementary file 2 — Expanded View Figures PDF [file EMMM-8-626-s002.pdf]

Expanded View Figures

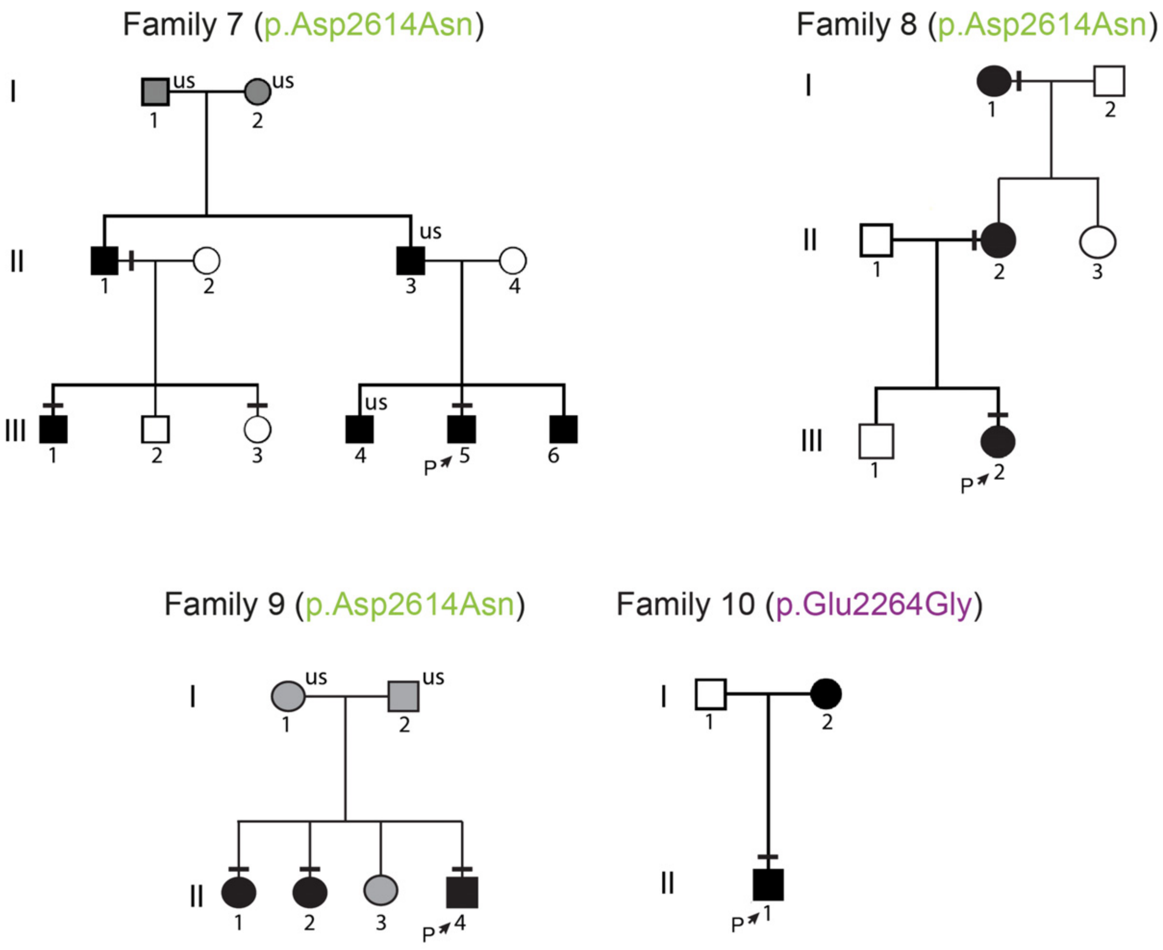

Figure EV1. Pedigrees of the families with two Further Potentially Pathogenic *IGSF10* mutations.
Squares indicate male family members; circles female family members. Black symbols represent clinically affected, gray symbols represent unknown phenotype, and clear symbols represent unaffected individuals. The arrow with "P" indicates the proband in each family, and "us" indicates unsequenced due to the lack of DNA from that individual. The mutation in each family is given next to the family number; a horizontal black line above an individual's symbol indicates they are heterozygous for that mutation as confirmed by either whole exome sequencing or Fluidigm array, and verified by Sanger sequencing.

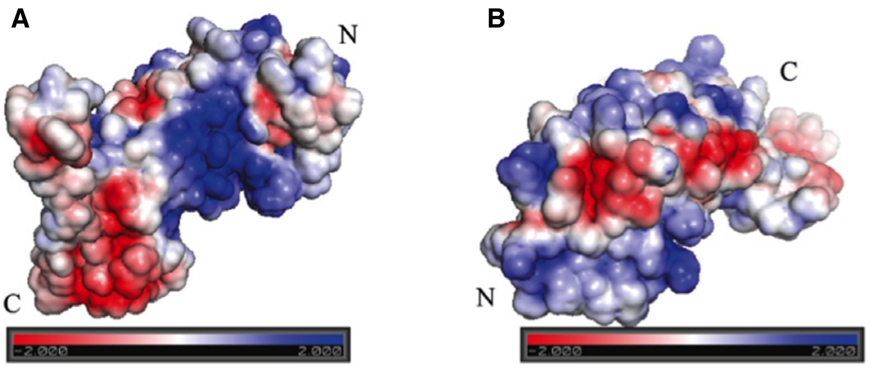

Figure EV2. Electrostatic map of LRR Region I.

- A Visualization of this region's electrostatic potential demonstrates the presence of a large positively charged patch on the inner concave surface
- B Visualization of this region's electrostatic potential demonstrates the presence of several interconnected negatively charged patches on the outer surface. These are likely to represent a protein–protein interaction site. LRR domains are usually extracellular.

 © 2016 The Authors

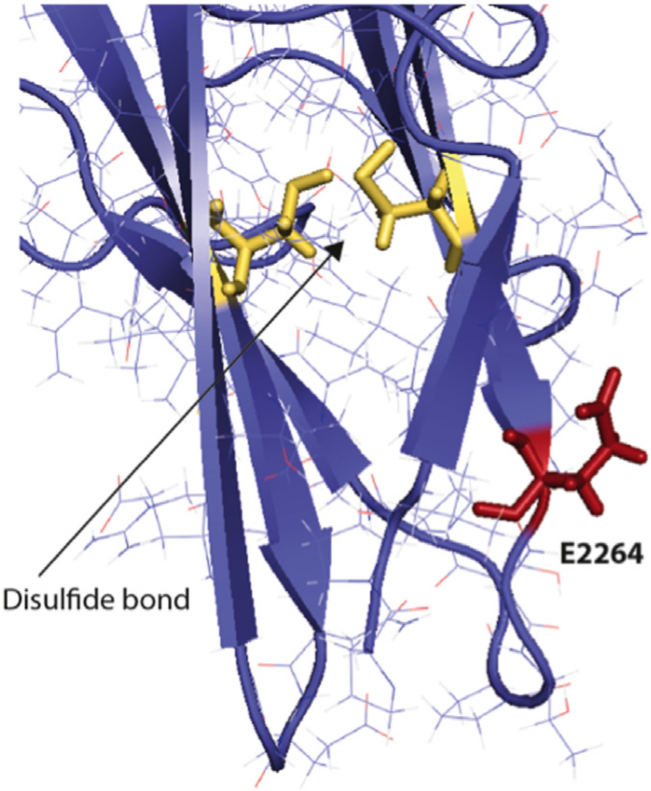

Figure EV3. Mutation p.Glu2264Gly.
Amino acid Glu2264 (E2264, in red) is localized on the surface of the Ig-like domain. Multiple sequence alignment shows that this position is occupied by negatively charged amino acids (the Uniprot Id of each homologue is indicated). Charge conservation among homologues and structural localization suggest that this amino acid may be involved in domain–domain interaction and substitution with the neutral Gly may well disrupt this possible interaction site. The disulfide bond in this Ig-like domain is shown in yellow.

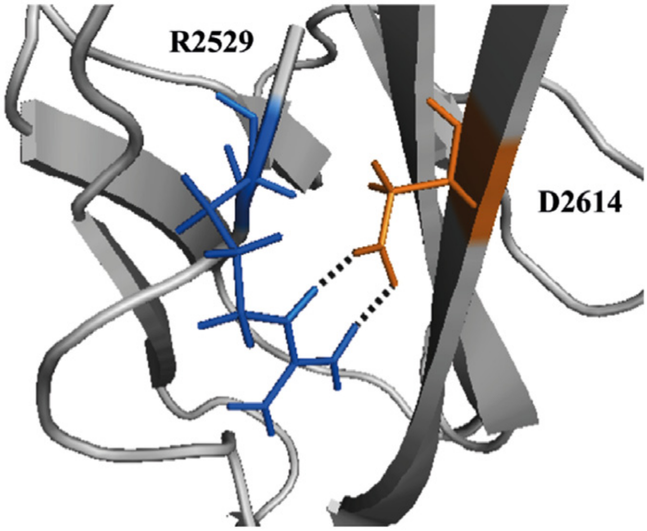

Figure EV4. Mutation p.Asp2614Asn.
Asp2614 (D2614, in orange) is predicted to form an intradomain a salt bridge with Arg2529 (R2529, in blue). Substitution of Asp2614 (D) with Asn (N) may weaken this interaction and destabilize the domain structure.

© 2016 The Authors

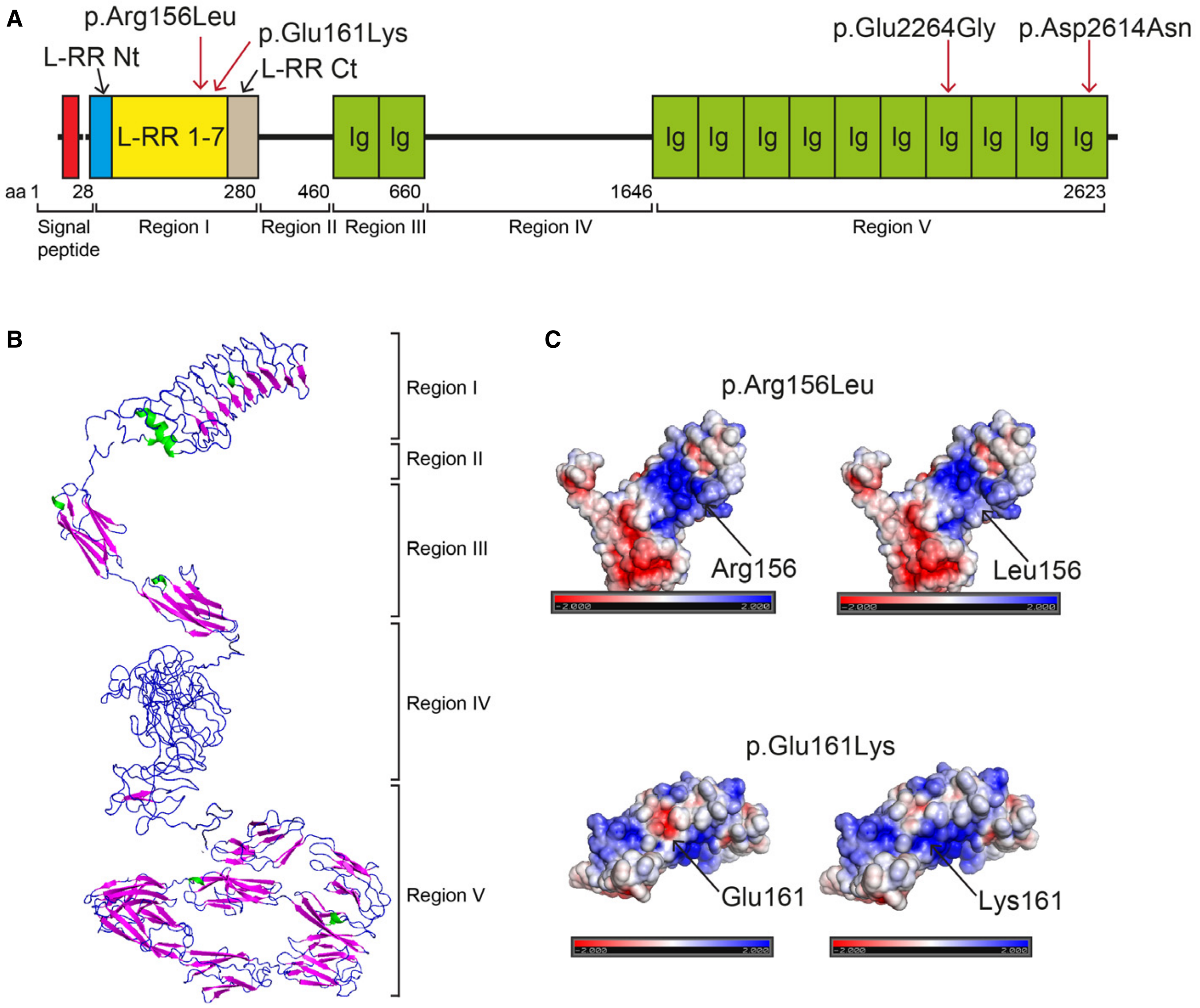

Figure EV5. IGSF10 protein structure and position of all four identified potentially pathogenic mutations.

A IGSF10 domains and mutations identified in the study. Region I contains leucine-rich repeats (LRR) 1–7 flanked by a LRR N-terminal (LRR Nt) and C-terminal (LRR Ct) cap. Region II is structurally disordered. Region III contains two Ig-like domains (Ig). Region IV is structurally disordered. Region V contains 10 Ig-like domains (Ig).

B Protein tertiary structure as predicted by *in silico* analysis.

C Electrostatic maps showing p.Arg156Leu and p.Glu161Lys mutations: p.Arg156Leu mutation—the electrostatic map shows a large positively charged region (displayed in blue) on the protein concave surface. Substitution of Arg with the neutrally charged Leu results in loss of part of the dark blue area; p.Glu161Lys—the electrostatic map shows a negatively charged patch (displayed in red) on the protein convex surface. Substitution of Glu with Lys results in loss of charge represented by loss of the dark red area.

 © 2016 The Authors
