# Supplementary figures and images for: IGSF10 mutations dysregulate gonadotropin‐releasing hormone neuronal migration resulting in delayed puberty
Source: EMBO Mol Med. 2016 Apr 13;8(6):626–42. doi: 10.15252/emmm.201606250 (PMC4888853; doi:10.15252/emmm.201606250)

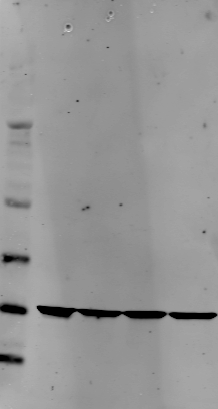

Supplement: Supplementary file 4 — Source Data for Figure 3 [file EMMM-8-626-s003.tiff]
